# Imprint of ancestral and modern threats in human mind – experience of fear, disgust, and anger

**DOI:** 10.3389/fpsyg.2024.1520224

**Published:** 2025-01-15

**Authors:** Eva Landová, Jakub Polák, Markéta Janovcová, Iveta Štolhoferová, Šárka Peterková, Aleksandra Chomik, Daniel Frynta

**Affiliations:** ^1^Department of Zoology, Faculty of Science, Charles University, Prague, Czechia; ^2^National Institute of Mental Health, Klecany, Czechia; ^3^Department of Psychology and Social Sciences, Ambis University, Prague, Czechia

**Keywords:** behavioral immune system, COVID-19, evolutionary psychology, fear module, pandemic

## Abstract

**Introduction:**

Threats to our survival are often posed by the environment in which humans have evolved or live today. Animal and human ancestors developed complex physiological and behavioral response systems to cope with two types of threats: immediate physical harm from predators or conspecifics, triggering fear, and the risk of infections from parasites and pathogens leading to the evolution of the behavioral immune system (BIS) with disgust as the key emotion. Here we ask whether the BIS has adapted to protect us from pandemic risks or poisoning by modern toxic substances.

**Methods:**

We have developed a survey comprised of 60 vignettes describing threats evoking fear and disgust belonging to one of the three main categories of threats: (1) ancestral, (2) modern, and (3) pandemic of airborne disease. Each vignette was evaluated on a 7-point Likert scale based on fear, disgust, and anger. Respondents also completed an assessment battery.

**Results:**

The results show that the strongest fear is triggered by modern threats (electricity, car accidents), while the highest disgust is evoked by ancient threats (body waste products, worms). Disgust does not respond to modern threat stimuli such as toxic substances or radioactivity as these evoke mainly fear and anger. A discriminant factor analysis classified nine out of 10 pandemic disgust vignettes into the ancestral disgust category, convincingly assigning the pandemic disgust threats to the ancestral type. Gender, age, and type of education were significant moderators of emotional responses across all threat categories.

**Discussion:**

Our study reveals that while fear is more context-dependent, particularly triggered by modern threats, disgust operates on an evolutionarily hardwired basis, making it less effective against contemporary risks. Furthermore, disgust experienced during a pandemic outbreak is more closely aligned with ancestral disgust-related threats tapping into evolutionary ancient survival circuits of the BIS. However, as disgust declines with age, the brain must adaptatively shift the emotional processing from disgust to fear to protect older adults from contamination risks. Finally, our study reveals that pandemic fear is better predicted by specific behaviors rather than general anxiety, suggesting a need for new assessments.

## 1 Introduction

Fear is a fundamental emotion serving as a critical adaptive mechanism that has allowed our ancestors to navigate and survive a world fraught with dangers (LeDoux, 2012). Various models exploring the cognitive, emotional, and physiological processes underlying fear have been developed to explain how these responses are triggered and maintained, particularly by ancestral and modern hazards, either imminent or abstract (Seligman, 1971; Öhman and Mineka, 2001; Poulton and Menzies, 2002; LeDoux, 2014).

Ancestral threats refer to the dangers present throughout much of human evolution, often linked to survival challenges faced by early humans. These include predation, interspecies competition, and environmental hazards like extreme weather conditions or natural disasters (Prokop, 2016; Ashley and Swick, 2019). Snakes are frequently cited as a primary ancestral threat evoking intensive fear. According to the latest estimates, more than 125,000 humans are killed by venomous snake bites annually (Afroz et al., 2024). Studies have shown that humans are biologically prepared to detect and respond to snakes more rapidly than to other stimuli, indicating a default attentional bias toward these creatures (Öhman et al., 2001; LoBue and DeLoache, 2008; cf. Zsido et al., 2024). Fear of snakes has been documented in infants as young as 5 months (Hoehl et al., 2017; Rakison, 2018; Bertels et al., 2020) and adults from distant cultures (Frynta et al., 2023). Moreover, attentional bias toward snakes is characteristic not only for humans but other non-human primates too, indicating that snake fear is evolutionarily ancient (Kawai and Koda, 2016; Masataka et al., 2018). Beyond specific animals, certain environmental factors, such as heights and darkness, also evoke fear responses (Prokop, 2016; Peléšková et al., 2024). For example, fear of heights may have developed as a survival strategy to prevent falls from dangerous elevations.

Certain ancestral threats continue to significantly impact mental health when an initially adaptive fear response becomes irrational and persistent due to the failure of neural regulatory mechanisms. Specific phobias, particularly ophidiophobia (fear of snakes) and acrophobia (fear of heights), are prevalent mental disorders that can significantly worsen individuals’ lives (Polák et al., 2016; Eaton et al., 2018). As societies transition toward modernization, the impact of ancestral threats on human behavior remains a critical area of study. The ability to differentiate between cues to threats in ancestral environments and those in the contemporary world is essential to avoid evolutionary mismatches that can lead to negative consequences (Shapouri et al., 2023).

However, fear is not the only basic emotion shaped by evolution to respond effectively to multiple risks in the environment. Similarly to the fear module (Öhman and Mineka, 2001), another psychophysiological, cognitive, and neural system in the human mind with disgust in its core has been proposed, the so-called behavioral immune system, which is activated to avoid potential pathogens and harmful substances (Schaller and Park, 2011).

Ancestral disgust elicitors can be categorized into several domains, primarily focusing on pathogen, sexual, and moral disgust (Tybur et al., 2009). Pathogen disgust is associated with stimuli that signal the presence of disease or contamination, such as rotting food, bodily fluids, or poor hygiene (Tybur and Lieberman, 2016). Sexual disgust reflects evolutionary adaptations related to reproductive strategies (Crosby et al., 2019). Finally, moral disgust is a more complex category that extends beyond physical threats to include social and ethical violations (e.g., cheating, stealing, or other forms of moral transgression that undermine social cohesion; Giner-Sorolla et al., 2018).

Research by Peléšková et al. (2024) highlights how ancestral disgust stimuli tend to group together, indicating a distinct response mechanism to threats that evoke feelings of repulsion and avoidance. Moreover, the study by Skolnick and Dzokoto (2013) comparing levels of disgust sensitivity and contamination between students from Ghana and the United States suggests that regions with a history of contagious disease threats exhibit higher levels of disgust. In the realm of cognitive processing, the study by Zhang et al. (2015) delves into the brain’s response to core and moral disgust pictures, indicating distinct neural processing mechanisms for different types of disgust-evoking stimuli. This suggests that the brain processes threats associated with disgust in a specialized manner. Moreover, the research by Van Hooff et al. (2013) on attentional biases toward disgust-evoking images highlights how such stimuli capture and hold attention, underscoring the salience of disgust-inducing threats in cognitive processing. All this research supports the evolutionary perspective put forth by Curtis et al. (2004) that human disgust likely evolved as a response to environmental objects representing infectious disease threats and potentially harmful substances.

Pandemic outbreaks presenting a specific type of threat have been a recurring theme throughout human history, shaping societies, economies, and health systems in profound ways. The historical record reveals a series of pandemic outbreaks that have left indelible marks on humanity, from the Black Death in the 14th century to the more recent COVID-19 pandemic (Barrett et al., 1998). The bubonic plague, caused by the bacterium *Yersinia pestis*, is one of the most notorious pandemics in history. The first major outbreak, known as the Justinian Plague, occurred in the 6th century, followed by the Black Death in the 14th century, which decimated approximately one-third of Europe’s population (Benedictow, 2021). Recent genetic studies have confirmed the presence of *Yersinia pestis* in remains from Black Death sites (Raoult et al., 2000; Drancourt et al., 2004). The plague’s impact extended beyond immediate health crises, as it led to significant social upheaval, labor shortages, and economic transformations across Europe (Sayed and Peng, 2021).

The Black Death serves as an example of a zoonotic disease, where an infectious pathogen is transmitted from a non-human host to humans, either directly or via a vector. In contrast, airborne diseases are transmitted through respiratory droplets or aerosols. The spread of these pathogens is heavily influenced by human mobility and the frequency of contact within and across populations (Tatem et al., 2012). The 1918 influenza pandemic, caused by the H1N1 virus, is one of the deadliest pandemics in history, resulting in an estimated 50 million deaths worldwide. This pandemic highlighted the importance of airborne transmission, as the virus spread rapidly in crowded urban areas, exacerbated by the conditions of World War I, where troop movements facilitated the spread of the virus across continents (Hoeven et al., 2009). The ability of influenza viruses to mutate and adapt to human hosts has been a consistent theme in the history of airborne pandemics, as seen in the emergence of new strains that can evade immunity and spread efficiently (Hu et al., 2020).

In 2002–2003, the outbreak of SARS (severe acute respiratory syndrome) marked a pivotal moment in understanding airborne transmission. The disease was primarily spread through respiratory droplets but also demonstrated airborne transmission in certain settings, such as hospitals (Beggs, 2003). This outbreak prompted a re-evaluation of infection control measures, particularly in healthcare settings, where airborne precautions became critical to prevent nosocomial infections. Recently, the coronavirus disease (COVID-19) pandemic has further emphasized the significance of airborne transmission of the virus (Yun et al., 2020; Chang et al., 2023). Studies have shown that SARS-CoV-2 can remain viable in aerosols for extended periods, leading to widespread transmission in various environments, including workplaces, schools, and public transport, i.e., enclosed spaces with poor ventilation (Dai and Zhao, 2022; Caracci, 2023).

The COVID-19 pandemic significantly elevated anxiety levels globally, driven by fears of infection, uncertainty, and social isolation. Studies have shown that pandemic-related anxiety is associated with heightened health-related concerns, avoidance behaviors, and stress over economic stability (Ahorsu et al., 2022). The Fear of COVID-19 Scale (FCV-19S), developed to measure such fears, highlighted that individuals with pre-existing mental health conditions and higher trait anxiety were particularly vulnerable (Taylor et al., 2020). These findings underscore the need for targeted psychological interventions during public health crises to mitigate anxiety and promote resilience.

In this regard, two distinct constructs of anxiety are often distinguished within clinical research reflecting different temporal and situational dynamics, i.e., trait and state anxiety. Trait anxiety refers to a stable, enduring predisposition to perceive a wide range of situations as threatening, linked to personality factors. High levels of trait anxiety are associated with heightened sensitivity to stress and a greater likelihood of experiencing state anxiety during stressful events, illustrating their interconnection (Endler and Kocovski, 2001). State anxiety, on the other hand, refers to a transient emotional condition characterized by heightened nervousness, tension, and apprehension in response to specific situations perceived as threatening. Unlike trait anxiety, state anxiety is temporary and fluctuates depending on external stressors (Spielberger et al., 1983).

We argue that pandemics of airborne diseases have emerged as a significant threat to human survival only in recent centuries. Throughout most of human evolutionary history, people lived in small, isolated groups with limited inter-group interaction, so only localized epidemics were expected (Weisdorf, 2005). This began to shift approximately 10,000 years ago with the rise of the first cities and the establishment of extensive trade networks (Cockburn, 1971). The rapid growth of the global human population and increased population density was necessary for the fast spread of infectious pathogens carried by air (Kakehashi and Yoshinaga, 1992). Therefore, from an evolutionary standpoint, pandemics of airborne diseases should not be regarded as an ancestral threat (see also Troisi, 2020).

The interconnectedness of the world today means that diseases can traverse borders with unprecedented speed, making effective management and containment more challenging as seen in recent outbreaks of diseases like COVID-19, Ebola, and SARS (Karlsen and Antonsen, 2023). This trend suggests that the frequency and impact of pandemics may continue to rise, necessitating a comprehensive understanding of their historical context to inform future public health strategies (Madhav et al., 2017).

Additionally, it has been suggested that since the beginning of the 20^th^ century, we have not been well adapted to effectively combat airborne diseases. For most of human history, airborne transmission was widely believed for many diseases (the so-called miasmatic paradigm) but this view was challenged by the rise of germ theory in the 19th century. Charles Chapin, a key figure in public health, helped shift the dominant belief to droplet/contact transmission in 1910, dismissing airborne transmission. This belief persisted until the 1960s when tuberculosis was proven to be airborne. The COVID-19 pandemic has renewed research, highlighting the importance of airborne transmission for many respiratory diseases (Jimenez et al., 2022).

Modern threats have largely emerged in the post-industrial era, with significant changes occurring in the last few 100 years. Modern (ontogenetic) threats are largely anthropogenic, i.e., they have emerged primarily because of human societal development and technological advancement. They can be physical, such as radiation exposure, toxicity from environmental pollutants, car accidents, and electrical hazards or psychological, such as stress or social challenges (Shapouri et al., 2023).

Car accidents represent a significant global public health issue, with mortality rates that have garnered attention from researchers and health organizations alike (Factor et al., 2010; Yousefzadeh-Chabok et al., 2016). According to the World Health Organization, approximately 1.19 million people died in 2023 due to road traffic accidents worldwide (World Health Organization, 2023). The distribution of these fatalities is not uniform across the globe. Developing countries bear a disproportionately high burden of road traffic deaths, often attributed to rapid motorization that outpaces infrastructure development Furthermore, the WHO has indicated that road traffic injuries are projected to become the fifth leading cause of death by 2030 if current trends continue.

Radiation emergencies are another type of modern danger that did not exist until the middle of the 20^th^ century. Nowadays, they have the potential to threaten human survival. In the immediate aftermath of the atomic bombings in August 1945, it is estimated that over 100,000 people died as a direct result of the blasts and acute radiation sickness (Jacob et al., 2010). Subsequent studies have suggested that the total number of deaths attributable to radiation exposure may be much higher when considering long-term effects (Douple et al., 2011; Yokota et al., 2017). The Chernobyl disaster in 1986 also resulted in significant mortality due to radiation exposure. Initial estimates suggested that around 31 people died from acute radiation syndrome shortly after the incident (Musa et al., 2019). However, long-term studies have indicated that thousands more may die from radiation-induced cancers and other health complications. Furthermore, the psychological effects of radiation fear, or radiophobia, can exacerbate the perceived health risks, leading to significant societal impacts that may overshadow the actual health risks posed by radiation (Lindberg, 2021).

In a previous study, we demonstrated that the strongest fear was elicited by modern threats (e.g., car accidents, electricity), while ancestral threats (e.g., body waste products, worms) evoked the highest levels of disgust. By contrast, disgust did not respond as strongly to modern risks like toxic substances, which mainly provoked fear and anger (Peléšková et al., 2024). Research also indicates that the human brain processes ancestral and modern threats differently, with evolutionary-threatening stimuli activating specific regions associated with threat detection and response, i.e., amygdala, thalamus, frontal gyrus, and fusiform gyrus. Modern threats, on the other hand, activated more the posterior cingulate and the parahippocampal gyrus (Dhum et al., 2017). Studies show that subcortical pathways may facilitate responses to ancestral threats, while cortical mechanisms may facilitate responses to modern threats (Vida and Behrmann, 2017). The amygdala, known for its role in fear processing, responds faster to evolutionary-relevant animate threats compared to equally threatening modern objects (Yang et al., 2012).

Gender differences in disgust sensitivity have been consistently documented, with women generally exhibiting higher levels of disgust than men across various contexts (Petrowski et al., 2010; Polák et al., 2019). This difference is thought to be rooted in evolutionary adaptations, where higher disgust sensitivity in women may have evolved to minimize fetal contamination during pregnancy (Kaňková et al., 2023). Gender differences extend to physiological responses as well. The research found that women exhibited greater electrodermal reactivity when exposed to disgust-inducing stimuli compared to men (Rohrmann et al., 2008). Women are also more likely to experience specific phobias and report higher levels of fear in response to certain stimuli, such as snakes or spiders (Rakison, 2009; Polák et al., 2016, 2020, 2022; Rádlová et al., 2020). This disparity may be attributed to a combination of biological predispositions and sociocultural factors. For example, women are more likely to believe in myths surrounding snakes, which may exacerbate their fear (Pinheiro et al., 2016).

Age also plays a critical role in the experience of fear and disgust. Research indicates that children as young as 2.5 years can express disgust and fear, suggesting that these emotions are fundamental to human experience and develop early in life (Stevenson et al., 2010). As individuals mature, the nature of their fears can evolve. For instance, while children may exhibit a more generalized fear of snakes and spiders, adults often display more specific phobias that can be linked to personal experiences or cultural narratives (LoBue et al., 2010; Rakison, 2022). Furthermore, the cognitive processing of fear-relevant stimuli appears to differ with age; younger individuals may rely more on instinctual responses, while older individuals may engage in more complex cognitive evaluations of threats (LoBue et al., 2010).

Fear of the pandemic among older adults is a significant concern, as older individuals perceive themselves at higher risk for illness, hospitalization, and death, leading to elevated levels of fear compared to younger groups (Caycho-Rodríguez et al., 2022). During the COVID-19 pandemic, older adults experienced intensified fears related to health, isolation, and uncertainty about the future (Losada-Baltar et al., 2021). This fear response is exacerbated by limited access to healthcare and social support, as well as mobility constraints, further amplifying their sense of vulnerability (Brooke and Jackson, 2020). While fear can promote caution and adherence to health measures, excessive fear may lead to negative psychological effects, such as stress, depression, and social withdrawal (Meng et al., 2020).

The temporal origins of perils to our survival vary significantly. The rise of technology, urbanization, and changes in social structures have introduced new challenges that our ancestral adaptations are not fully equipped to handle (Diamond, 1999). The concept of evolutionary mismatch highlights how certain modern threats might be ill-suited to our ancestral adaptations (Williams, 1966; Lieberman, 2014). This research aims to explore whether the evolutionary survival circuits (fear module and behavioral immune system), which evolved to protect us against dangers like predators and diseases, are still effective in managing contemporary threats including pandemics. By examining fear, disgust, and anger, we seek to understand whether our psychological responses to a pandemic are rooted in deep evolutionary mechanisms or if they represent an adaptation to the complex challenges of the modern era. Additionally, the study will examine how these emotional responses vary across different demographic groups, with a particular focus on age and gender. Understanding whether younger or older individuals, as well as different genders, are more responsive to either ancestral, modern, or pandemic threats could reveal how evolutionary and individual factors intersect in shaping our reactions to danger.

Finally, fear is widely recognized as stimulus-specific, meaning that individuals exhibit a predisposition to fear certain stimuli over others based on evolutionary and experiential factors. Furthermore, there is considerable interindividual variability, where different individuals display varying degrees of fear toward a given stimulus. Such differences underscore the importance of accounting for individual characteristics - such as past experiences and personality traits including emotional reactivity and sensitivity - when examining fear responses to ancestral, modern, and pandemic-related threats. Accounting for these variations is essential for accurately capturing broader emotional response patterns across different threat categories.

We will investigate these questions:

- How strong are the emotions of fear, disgust, and anger in response to different types of ancestral and modern threats with special regard to a pandemic of airborne diseases?
- How do the respondents’ characteristics (sex, age, education) influence the subjective emotional evaluation of these threats?
- How does the respondents’ emotional reactivity (e.g., fear of snakes, disgust propensity, stress of COVID-19, or anxiety) influence the subjective evaluation of these threats?

## 2 Materials and methods

### 2.1 Respondents

**Figure 1:**
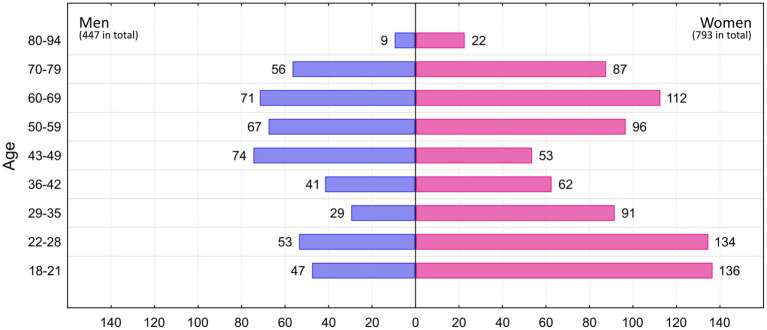
Number of men and women respondents in each age category.

Previously, 629 respondents (463 women, 166 men) participated in an experiment conducted by Peléšková et al. (2024) in which they rated only vignettes according to fear, disgust, and anger. For the present study, they were again approached to additionally complete a battery of psychological questionnaires (see specifically below). In addition, 611 new respondents (330 women, 281 men) participated in this study and completed both parts of the experiment. The new respondents were selected with respect to the characteristics of respondents from the previous study to fill in missing numbers in various categories (e.g., men, older persons, different type of education than biological). Thus, our total sample of 1,240 respondents (793 women and 447 men) included people aged 18–94 years (mean 45 years). For a detailed distribution of respondents by age group and gender, see Figure 1.

All participants were from Central Europe and completed the experiment in Czech. The sample was recruited from various colleges, including a university of the third age, along with their relatives, which broadened the educational diversity of the participants. Students are a commonly utilized population in psychological research due to their willingness to participate and their ability to handle more complex experimental designs. Including their family members and friends expanded the sample to individuals without higher education but with strong motivation to complete the study. Specifically, the educational background of the respondents included 135 individuals with primary education, 422 with secondary education, and 683 with a university degree. As noted by Peléšková et al. (2024), individuals with a biological education may differ in their evaluation of certain stimuli or situations compared to those with other educational backgrounds. In this study, 243 respondents had a biological education (broadly defined), 127 had an agricultural education, and 93 had a medical education, while 777 participants reported other educational fields (technical, social sciences, economics, general, etc.). A complete list of respondents with detailed socio-demographic characteristics is provided in Supplementary Table S1.

### 2.2 Vignettes

In this study, we employed 60 vignettes depicting various potentially dangerous situations, previously published in Peléšková et al. (2024). The vignettes were categorized into six groups: ancestral fear (AF), ancestral disgust (AD), modern fear (MF), modern disgust (MD), pandemic fear (PF), and pandemic disgust (PD). For the exact wording of the vignettes and their categorization, refer to Supplementary Table S2. Respondents rated each vignette based on their perceived levels of fear, disgust, and anger.

### 2.3 Assessment

Each respondent provided basic socio-demographic information, including gender, age, and educational background, followed by a series of psychological questionnaires and self-reported evaluations of specific stimuli. The selected questionnaires were designed to assess respondents’ general sensitivity to various situations and specific stimuli. To address pandemic-related threats, questionnaires specifically focused on the COVID-19 pandemic were included. General assessments targeted anxiety and disgust sensitivity, with the State–Trait Anxiety Inventory-X2 (STAI-X2; Spielberger et al., 1983; Heretik et al., 2009) used to measure trait anxiety, the pathogen and moral disgust subscales of the Three-Domain Disgust Scale (TDDS; Tybur et al., 2009) to measure disgust sensitivity, and the abbreviated Snake Questionnaire (SNAQ-12; Zsido et al., 2018; Polák et al., 2020) to assess fear of snakes. Additionally, three questionnaires were administered to evaluate respondents’ emotions and behaviors during and after the COVID-19 pandemic: The Fear of COVID-19 Scale (FCV-19S; Ahorsu et al., 2022; Remr, 2023), The Coronavirus Safety Behaviors Scale (CSBS; Wheaton et al., 2012; Blakey et al., 2015) assessing retrospective pandemic behaviors, and The COVID Stress Scales (C-19SS; Taylor et al., 2020) measuring pandemic-related fears and stress during the first wave of COVID-19 (retrospective).

Respondents also rated their levels of fear and disgust for specific stimuli, including snakes, heights, vehicles, electricity, radioactivity, and toxic chemicals, using a 7-point Likert scale. Since tailored questionnaires assessing both fear and disgust for these stimuli are scarce, and administering additional assessments would exceed respondents’ capacity, Likert scale ratings were deemed sufficient to capture reliable data on emotional responses to these threats. Anger was excluded from these ratings, as evaluating it without a specific contextual situation could lead to misleading results. A summary of final scores for all questionnaires and self-reported fear and disgust ratings of stimuli is presented in Supplementary Table S1. Raw data for vignette ratings of fear, disgust, and anger are provided in Supplementary Tables S3–S5, respectively.

### 2.4 Procedure

Testing was conducted online and via pen-and-paper between October 2022 and May 2024. Each respondent first provided basic socio-demographic information, including age, gender, and educational background. Subsequently, participants rated each vignette on a 7-point Likert scale, assessing the perceived levels of fear, disgust, and anger (1 = not at all, 7 = extremely strong). Verbal stimuli, such as snakes, heights, and transport vehicles, were also rated on a 7-point Likert scale for fear and disgust. There was no time limit for completing the experiment. Older respondents received assistance when needed, particularly with reading the vignettes, and their responses were recorded by the experimenters based on their instructions.

### 2.5 Ethical note

This study was carried out following the approval of the Ethical Committees of Charles University, Faculty of Science (approval no. 2021/02, granted on 14 April 2021) and National Institute of Mental Health (no. 91/21, granted 31 March 2021) and following the Declaration of Helsinki. All subjects provided their informed consent with participation in the study and personal data processing.

### 2.6 Statistical analysis

Raw data were used for analyses wherever applicable, including vignette ratings, self-reported responses to various stimuli, and demographic variables such as age and gender (male and female; no other genders were reported in this dataset). Educational levels were categorized into three groups: primary, secondary (high school), and tertiary (university). The field of education was classified into seven categories: biological, agricultural, medical, technical, economic, social sciences, and general. Questionnaire data were analyzed using final scores, with subscale scores (e.g., pathogen and moral disgust from the Three-Domain Disgust Scale) treated separately. Agreement among respondents regarding fear, disgust, and anger ratings for vignettes was assessed using Kendall’s coefficient of concordance (R, irr package; R Core Team, 2019; Gamer et al., 2019).

To explore the similarity patterns of elicited emotions across different threat categories, discriminant functional analysis (DFA) was applied. Each vignette was characterized by three variables: average ratings of fear, disgust, and anger, forming a multidimensional space. All 60 vignettes were included in the analysis, with four custom-defined categories: ancestral fear, ancestral disgust, modern fear, and modern disgust. First, we evaluated the accuracy of classification by determining how well vignettes matched their predefined categories. We then used DFA to classify pandemic-related threats. To further investigate the contributions of each emotional response to the classification of vignettes, we conducted canonical variate analysis (CVA). Both analyses were performed in Statistica (StatSoft, Inc., 2014).

To assess the effects of threat type, gender, age, and education on fear, disgust, and anger ratings, we employed cumulative link mixed models (CLMM) using RStudio (R Core Team, 2023), with the ordinal package (Christensen, 2023), which is specifically designed for ordinal dependent variables such as Likert-scale ratings. The model converts raw ratings into probabilities for each category (1–7) using a logit function. The output includes threshold coefficients that indicate the cumulative probabilities for each rating category. For instance, if the threshold coefficient for 3|4 is 0.65, the probability of receiving a rating of 3 or lower is 65%. These cumulative probabilities can also be transformed into simple probabilities for individual categories, if necessary. The models also evaluate the effects of various factors on the threshold coefficients. On the logit scale, the effects are linear, meaning all threshold coefficients increase or decrease by the same magnitude. However, on the probability scale, the effects are non-linear, as an increase in the probability of a “7” rating requires a decrease in one or more other ratings. A Likelihood Ratio Test was used to compare full and reduced models (with and without the factor of interest) to determine whether a given factor significantly influenced the results. To control for multiple comparisons, we applied a Bonferroni correction, adjusting the significance level to *α* = 0.05/21 ≈ 0.00238.

Redundancy analysis (RDA) was employed to identify constrained gradients of variability in fear, disgust, and anger ratings. RDA allows for multivariate analysis of relationships between respondents’ characteristics, questionnaire scores, and vignette ratings. Statistical significance was determined through permutation tests. The raw data for vignette ratings by fear, socio-demographic characteristics of the respondents, final scores from all questionnaires, and self-reported fear of verbal stimuli were used to analyze the relationship between the perceived fear and individual characteristics. Similar procedures were followed for the analysis of disgust and anger, with raw data of vignette ratings by disgust or anger, respondents’ characteristics, final scores from all questionnaires, and self-reported disgust from the verbal stimuli used to investigate the triggers of these emotions. All analyses were conducted using R, with the vegan and MASS packages (Venables and Ripley, 2002; Oksanen et al., 2020).

## 3 Results

### 3.1 Agreement among respondents

Vignette ratings by all respondents included in this study showed a high level of agreement, with Kendall’s W 0.515 for fear, 0.409 for disgust, and 0.308 for anger. A similarly high agreement rate was found in a previous study by Peléšková et al. (2024). Interestingly, the highest agreement in fear ratings was for the modern vignettes (0.414 and 0.382), in disgust ratings for the pandemic vignettes (0.222 and 0.218), and in anger ratings for the ancestral vignettes (0.332 and 0.196). Detailed results can be found in Supplementary Table S6.

### 3.2 Pattern of fear, disgust, and anger in response to ancestral, modern, and pandemic threats

We used the discriminant functional analysis (DFA) to classify individual vignettes into four predefined categories: ancestral fear, ancestral disgust, modern fear, and modern disgust. The analysis had an overall success rate of 87.5%. Interestingly, six pandemic fear vignettes were classified into the ancestral and four remaining vignettes into the modern fear category, leaving the classification of pandemic fear threats inconclusive. On the other hand, nine out of 10 pandemic disgust vignettes were classified into the ancestral disgust category, convincingly assigning the pandemic disgust threats to the ancestral type of threats (detailed results are shown in Table 1).

**Table 1:** Classification of individual vignettes into the ancestral and modern types of threat.

Predefined category	Number of vignettes	Predicted classification				Success rate
		AF	AD	MF	MD	
Ancestral fear (AF)	10	8	0	2	0	80%
Ancestral disgust (AD)	10	0	10	0	0	100%
Modern fear (MF)	10	2	0	8	0	80%
Modern disgust (MD)	10	0	0	1	9	90%
Pandemic fear (PF)	10	6	0	4	0	$$
Pandemic disgust (PD)	10	0	9	0	1	$$
Total	60	16	19	14	10	87.5%

The analysis was asked to classify individual vignettes into four groups aligning with ancestral fear, ancestral disgust, modern fear, and modern disgust categories (predicted classification). $$ The success rate cannot be assessed as the analysis was not allowed to classify the vignettes into pandemic categories.

**Figure 2:**
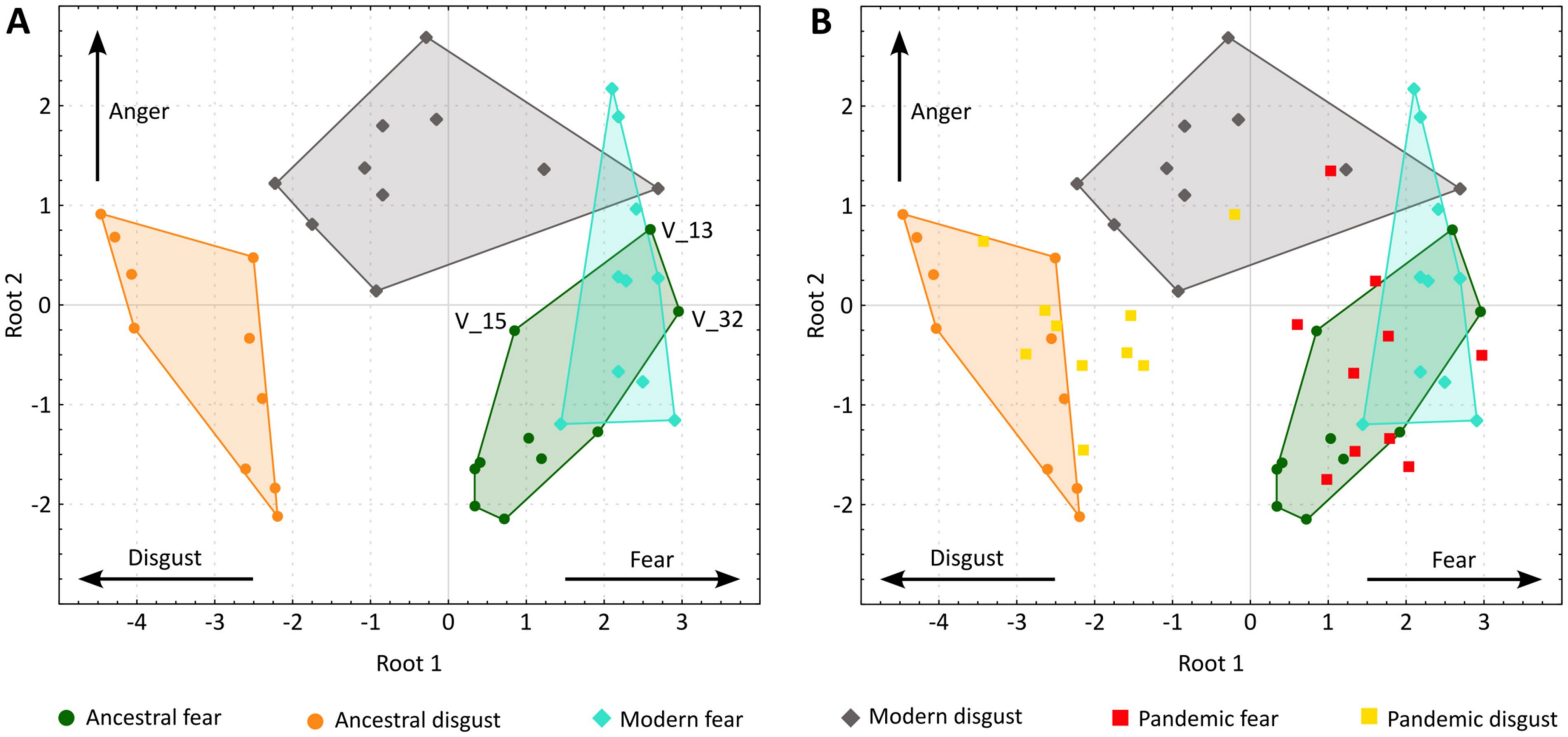
Canonical scores for each vignette. **(A)** Vignettes evoking non-pandemic threats. **(B)** All 60 vignettes, including the pandemic-related ones. Vignettes representing ancestral fear, ancestral disgust, modern fear, and modern disgust are enclosed by the smallest convex polygon to better visualize the overlaps between the threat categories. The labeled vignettes (V_13, V_15, V_32) stand out from other ancestral fear vignettes due to relatively high anger ratings. Although these vignettes focus on the fear of snakes or heights, the dangerous situation was caused by a person, e.g., V_13: From the sidewalk, I see a small child climbing on the balcony railing on the 10th floor.

To further assess how the intensities of each investigated emotion contribute to the classification of individual vignettes, we performed the canonical variate analysis (CVA). The analysis supported two roots: root 1 (eigenvalue 4.65, *p* < 0.0001) correlated with the fear (factor loading 0.60) and disgust ratings (factor loading −0.88), while root 2 (eigenvalue 0.92, *p* < 0.0001) correlated with the anger rating (factor loading 0.98). To visualize the results, we plotted the canonical scores for each vignette (Figure 2).

**Figure 3:**
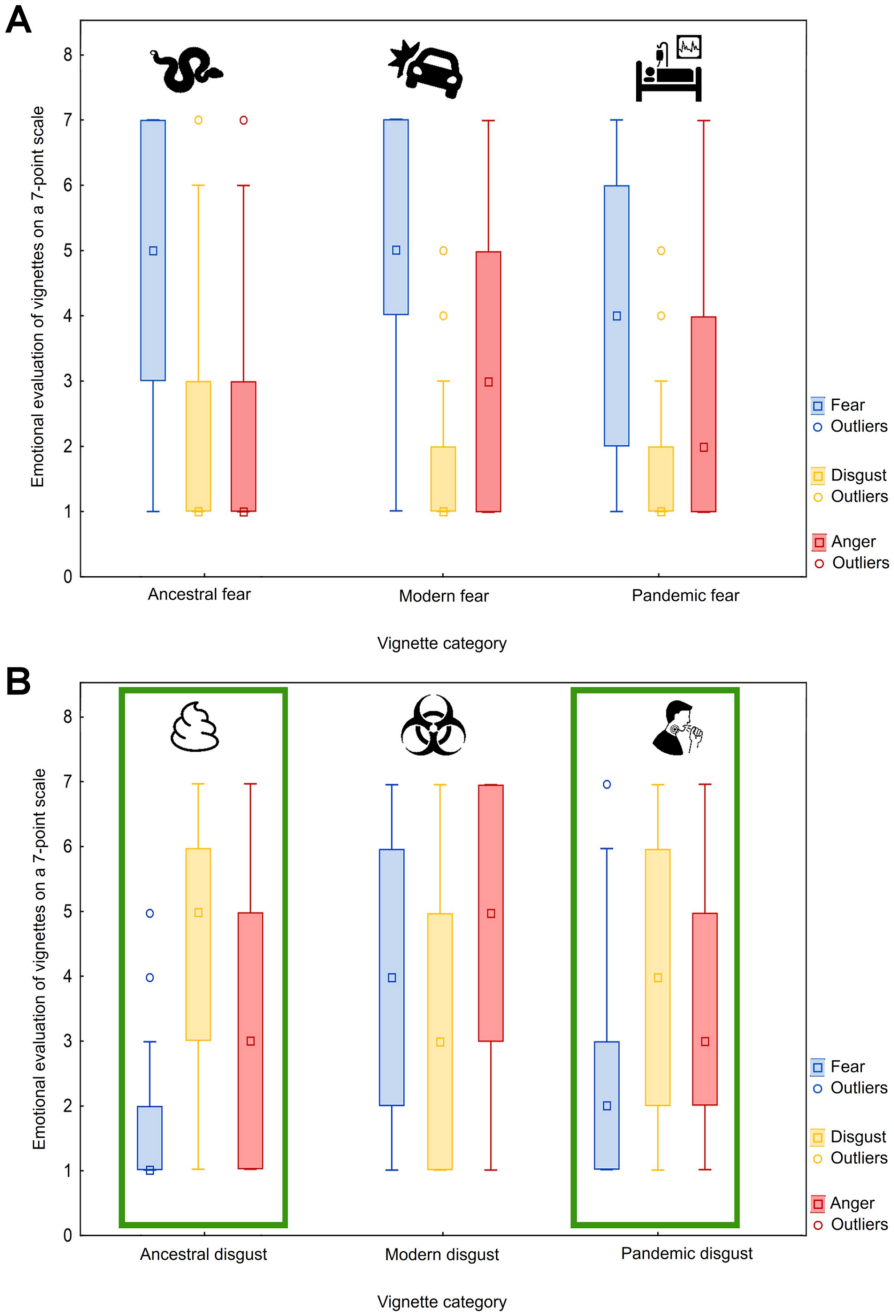
Boxplots of emotional evaluation of vignettes according to fear, disgust, and anger. Box plots graphically depict the ratings of the fear **(A)** and disgust **(B)** vignette categories according to the three dimensions (fear, disgust, and anger). The green box highlights the vignette categories that most closely match the pandemic vignette category (relevant only for pandemic disgust) in their results. Median (middle point), lower and upper quartiles (box range), and non-outlier minimum and maximum values (whiskers) are provided together with outlier points.

Finally, we tested whether the type of threat affects the intensity of evoked emotions. For all three emotions, the effect was highly significant (*p* < 0.0001). Modern fear (MF) threats evoked the highest fear, followed by ancestral fear threats (AF; MF-AF: coefficient = −0.44, Z-value = −18.92, *p*-value <0.0001), while pandemic fear threats evoked the lowest fear (PF; MF-PF: coefficient = −1.06, Z-value = −44.95, *p*-value <0.0001; AF-PF: coefficient = −0.61, *Z*-value = −26.71, *p*-value <0.0001). Contrary, ancestral disgust (AD) threats evoked the highest disgust, followed by pandemic disgust threats (PD; AD-PD: coefficient = −0.61, *Z*-value = −26.75, *p*-value <0.0001) while the modern disgust threats evoked the lowest disgust (MD; AD-MD: coefficient = −0.46, *Z*-value = −20.23, *p*-value <0.0001; PD-MD: coefficient = −0.61, Z-value = −26.71, *p*-value <0.0001). Finally, the greatest anger was elicited by modern disgust threats, followed by pandemic disgust and ancestral disgust threats (MD-PD: coefficient = −0.90, Z-value = −39.21, *p*-value <0.0001; PD-AD: coefficient = −0.01, *Z*-value = −0.60, *p*-value = 0.551), then modern fear threats (AD-MF: coefficient = −0.38, *Z*-value = −16.09, *p*-value <0.0001), pandemic fear threats (MF-PF: coefficient = −0.57, *Z*-value = −23.45, *p*-value <0.0001), and lastly ancestral fear threats evoked that the lowest anger (PF-AF: coefficient = −0.53, *Z*-value = −20.86, *p*-value <0.0001; see Figure 3 for visualization).

### 3.3 Role of gender, age, and education in emotional response to ancestral, modern, and pandemic threats

**Figure 4:**
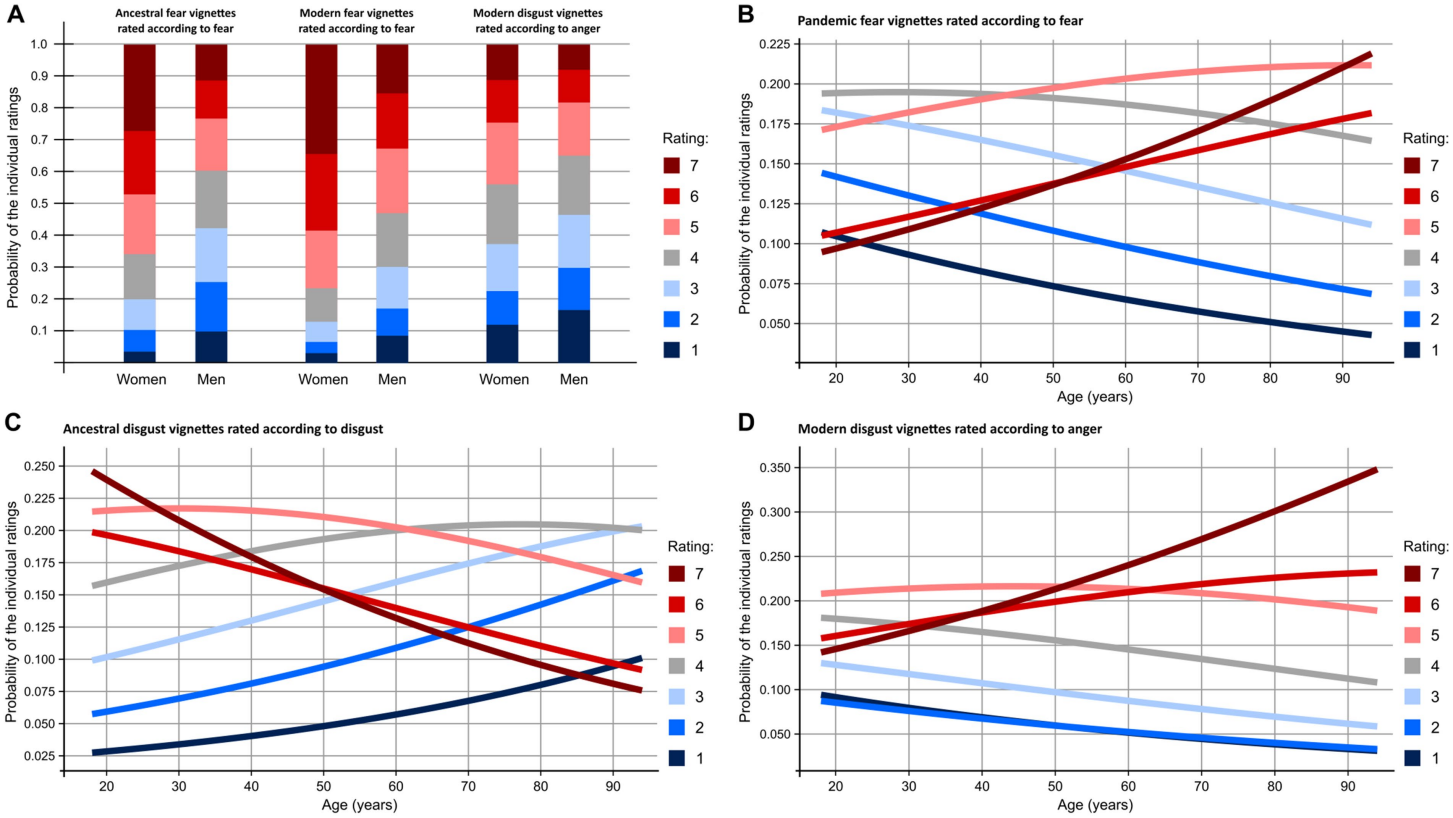
Probability of individual ratings as a function of respondents’ sex **(A)** and age **(B–D)**. While women‘s ratings were steadily equal to or higher than men‘s, the effect of sex was more varied. Disgust ratings of ancestral disgust stimuli declined with age but fear of pandemic fear threats as well as anger of modern disgust threats increased. Notice the complete shift in reporting on elicited disgust: While ancestral disgust threats are perceived as extremely disgusting (rate 7) by almost a quarter of 20-year-olds, the same rate is the one least likely (about 8%) to be given by 90-year-olds.

To assess the effect of gender, age, and education type on fear, disgust, and anger ratings of individual types of vignettes, we utilized cumulative link mixed models (CLMM). Education was divided into seven categories: agricultural, biological, economic, medical, humanities, technical, and other. Simplified results are shown in Table 2 and Figure 4, the full results are in Supplementary Tables S7, S8.

**Table 2:** Simplified results for the effect of gender, age, and education type on fear, disgust, and anger ratings of all vignette types.

Vignette type	Tested factor	Rating according to elicited		
		Fear	Disgust	Anger
Ancestral fear	Gender	F > M* ***Z = -11.5, p < 0.0001***	F = M Z = -0.5, *p* = 0.6529	F = M Z = -0.7, *p* = 0. 4,780
	Age	—	—	—
		Z = 2.7, *p* = 0.0072	Z = 0.8, *p* = 0.4210	Z = 1.1, *p* = 0.2815
	Education	***Lowest: biological***	***Highest: other Lowest: biological***	***Highest: other lowest: biological***
Modern fear	Gender	F > M*	$$	F = M
		***Z = -11.8, p < 0.0001***		Z = -0.3, *p* = 0.7672
	Age	—	$$	—
		Z = 0.3, *p* = 0.7980		Z = -1.7, *p* = 0.0954
	Education	—	$$	***Lowest: biological***
Pandemic fear	Gender	***F > M***	F = M	F = M
		***Z = -7.0, p < 0.0001***	Z = 1.9, *p* = 0.0553	Z = -0.9, *p* = 0.3675
	Age	↗*	—	—
		***Z = 4.8, p < 0.0001***	Z = -1.0, *p* = 0.3186	Z = -0.1, *p* = 0.9320
	Education	—	***Highest: other Lowest: biological***	***Highest: other Lowest: biological***
Ancestral disgust	Gender	F = M	***F > M***	F = M
		Z = -1.6, *p* = 0.1182	***Z = -9.4, p < 0.0001***	Z = -2.6, *p* = 0.0100
	Age	—	↘*	—
		Z = 0.5, *p* = 0.6400	***Z = -7.4, p < 0.0001***	*Z = -7.4, p = 0.0027*
	Education	***Lowest: biological***	—	***Highest: other***
Modern disgust	Gender	***F > M***	F = M	F > M*
		***Z = -6.2, p < 0.0001***	Z = -1.7, *p* = 0.0914	***Z = -3.8, p = 0.0001***
	Age	—	↘	↗*
		Z = 0.2, *p* = 0.8512	***Z = -3.3, p = 0.0008***	***Z = 3.6, p = 0.0004***
	Education	—	***Highest: other*** ***Lowest: biological***	—
Pandemic disgust	Gender	$$	***F > M*** ***Z = -7.3, P < 0.0001***	***F > M*** ***Z = -3.9, p < 0.0001***
	Age	$$	— Z = 1.3, *p* = 0.2112	— Z = 2.8, *p* = 0.0050
	Education	$$	—	—

Gender: F > M – women gave higher ratings than men, F = M – no significant difference between ratings of men and women. Age: ↗ – older participants gave higher ratings than younger ones, ↘ – older participants gave lower ratings than younger ones, — – participants’ age did not significantly affect their ratings. Education: education types with significantly higher and/or lower ratings than at least three other types are listed. — – participants’ education type did not significantly affect their ratings. No statistics are provided for the effect of education because the matrix would not fit this simplified table. Z – Z-value, P – P-value, significant results (*p* ≤ 0.0024) are in bold. * – the result is visualized in Figure 4; $$ – the model failed to converge due to insufficient data variability within some factor levels.

### 3.4 Variability of fear, disgust, and anger evaluation explained by individual sensitivity

**Figure 5:**
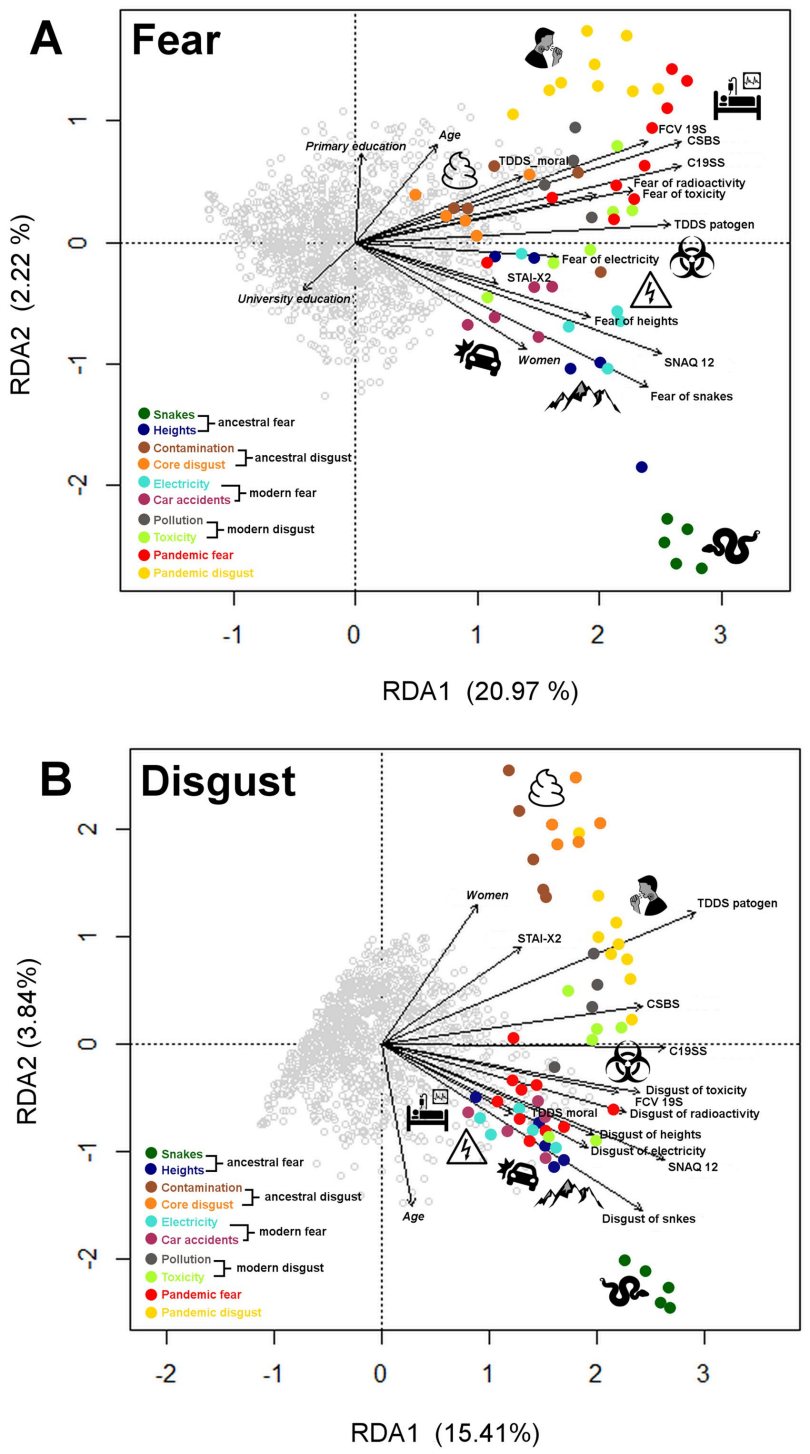
Graphical output from the redundancy analysis (RDA) for fear **(A)** and disgust **(B)** vignette ratings. The grey points in the graphs represent individual respondents, the colored points represent individual vignettes, and the colors correspond to the categories into which the vignettes are categorized (see the legend in each graph). Only statistically significant variables (*p* < 0.05) in the optimal model are shown. Data from vignette ratings by fear/disgust were included as explanatory variables in the models. The explained variables were (1) respondents’ socio-demographic characteristics (gender, age, and educational level - italics in the graphs); (2) general fear/disgust aroused by different categories of stimuli rated on a 7-point Likert scale (in the graphs the variables named “Fear of.…”/“Disgust of…”); (3) assessments: State–Trait Anxiety Inventory-X2 (STAI-X2); Three-Domain Disgust Scale, specifically the pathogen disgust (TDDS pathogen) and moral disgust (TDDS moral) subscale; Snake Questionnaire-12 (SNAQ-12); Fear of COVID-19 (FCV-19); Coronavirus Safety Behaviors Scale (CSBS); and COVID Stress Scales (C19SS).

The RDA model of fear ratings as a response variable explained 27.23% of the total variability. We then performed a permutation test (number of permutations = 20,000) to confirm the significance of each of the independent variables (constraints) in a sequential (‘type I’) test. All the analyzed explanatory variables were statistically significant at *p* < 0.05, those with the most significant effect were the instruments assessing behaviors during the pandemic (CSBS) and self-reported fear of snakes and toxicity (see Figure 5A for visualization of the RDA results and Supplementary Table S9 for more details). The first multivariate axis (RDA1) with an eigenvalue of 37.29 and 20.97% of explained variability is mostly associated with the respondent’s general emotional sensitivity. The second multivariate axis (RDA2) with an eigenvalue of 3.87 and 2.17% of explained variability could be interpreted as a difference among specific fear categories.

As for disgust ratings, the RDA model explained 22.85% of the total variability. Based on the permutation sequential (‘type I’) test, all the analyzed explanatory variables were statistically significant at *p* < 0.05. Variables with the most significant effect on disgust ratings were the pathogen disgust subscale of TDDS and self-reported disgust of snakes and toxicity (see Figure 5B for visualization of the RDA results and Supplementary Table S9 for more details). The RDA1 axis with an eigenvalue of 28.85 and 14.48% of explained variability pertains to disgust sensitivity (associated with snakes, pathogens, and toxic substances). The RDA2 axis with an eigenvalue of 7.21 and 3.87% of explained variability is associated with age (negatively correlated with reported disgust) and gender (women feeling more disgusted by the vignettes).

Finally, the RDA model for anger ratings explained only 16.55% of the full variability. Again, all the explanatory variables in the model had a significant effect on *p* < 0.05; the most significant were the pathogen disgust subscale of TDDS, self-reported disgust of toxicity, and stress of covid-19 measured by the C-19SS scale (see Supplementary Figure S1 for visualization of the RDA results and Supplementary Table S9 for more details). The RDA1 axis with an eigenvalue of 23.30 and 10.24% of explained variability could be interpreted as several domains of fear or disgust sensitivity (pathogen disgust, disgust of pathogens, and stress in response to the COVID-19 pandemic). The RDA2 axis with an eigenvalue of 2.19 and only 0.96% proved non-interpretable.

## 4 Discussion

### 4.1 Pattern of fear, disgust, and anger in response to ancestral, modern, and pandemic threats

Fear, disgust, and anger evaluations were compared across ancestral, modern, and pandemic-related threats. The results reveal a hierarchy in emotional responses, with modern fear stimuli eliciting the highest levels of fear, followed by ancestral and pandemic fear stimuli. Ancestral disgust elicitors triggered the strongest disgust, with pandemic and modern disgust triggers following. These results align with the findings reported in our previous study (Peléšková et al., 2024), providing further validation of the initial observations. The following sections present novel findings.

Interestingly, anger was most pronounced in response to modern disgust stimuli (such as pollution and radioactivity), followed by pandemic and ancestral threats. This finding corroborates previous research on the interaction between disgust and anger. Some studies suggest that disgust can fuel moral anger when it comes to violations of purity or contamination norms (Rozin et al., 1999; Hutcherson and Gross, 2011). In scenarios where a violation of a socially accepted moral code occurred (e.g., a dangerous snake escaped due to the negligence of its owner), our respondents reported experiencing anger rather than disgust. Furthermore, anger directed at pandemic-related threats, particularly those involving perceived negligence or harm such as the mishandling of public health measures, has been reported in other studies of pandemic emotions (Renström and Bäck, 2021). This may explain why pandemic disgust responses were also associated with elevated anger.

Our primary objective was to investigate the patterns of three specific emotional responses - fear, disgust, and anger - and to determine which type of threat the pandemic outbreak most closely resembles. The discriminant functional analysis (DFA) provided valuable insights into the categorization of emotional responses elicited by pandemic-related vignettes. The overall classification success rate of 87.5% demonstrates the robustness of the method in distinguishing between ancestral and modern emotional responses.

Specifically, the results highlight that pandemic-related disgust, a prominent emotional reaction during the COVID-19 pandemic, is more closely aligned with ancestral disgust threats, as nine out of ten pandemic disgust vignettes were classified under this category. This suggests that the threat of airborne pathogens like COVID-19 may tap into similar deeply rooted evolutionary circuits designed to protect against contamination and infection, paralleling ancient fears of disease transmission. The strong disgust responses to pandemic threats highlight the relevance of this evolved system in contemporary contexts, where the fear of contagion remains salient.

Our results emphasize the evolutionary basis of disgust as a part of the behavioral immune system, a defense mechanism against pathogens (Schaller and Park, 2011) and reinforce the notion that pandemics are perceived as an ancestral disgust threat, likely because of their direct link to disease, bodily fluids, and contamination risks. The clear assignment of pandemic disgust to the ancestral category contrasts with modern disgust, which tends to be triggered by more abstract or socially constructed threats, such as moral violations (Tybur et al., 2009).

The classification of pandemic fear, however, was less straightforward. While six pandemic fear vignettes were classified under ancestral fear and four under modern fear, this split suggests that pandemic fear is not as easily mapped onto a single category of threat. It suggests that pandemic fear includes some ancestral component related to physical danger and an immediate threat to survival, however, the classification of four pandemic fear vignettes into the modern fear category suggests that contemporary fears, such as economic insecurity or social isolation, are also salient in the context of pandemics. This dual classification reflects the multifaceted nature of fear in modern pandemics, where both ancient and novel threats coalesce.

Previous studies have consistently shown that evolutionarily fixed ancestral threats elicit the greatest fear responses. However, our findings suggest the contrary, that is the modern threats trigger the most pronounced fear. Specifically, contemporary dangers such as car accidents, pollution, and radiation - products of technological advancements and urbanization – elicit fear due to their immediate, life-threatening nature (Hasegawa et al., 2015). Furthermore, modern threats, particularly those related to pollution and toxicity, evoke fear not only because of their direct impacts but also due to their latent, cumulative effects. Although these modern dangers are less tangible than ancestral threats like predators, they may be perceived as more threatening because of their omnipresent and often uncontrollable nature.

Studies suggest that while ancestral threats activate more automatic, subcortical responses (such as the amygdala’s rapid response to animate threats), modern threats may engage more deliberative, complex cortical pathways that contribute to the heightened fear responses observed in this study (Vida and Behrmann, 2017). Consequently, fear responses to modern threats also involve a broader contextual assessment, including the identification of the threat and potential culpability. In contrast, responses to ancestral threats, such as snakes or heights, tend to lack this broader contextual evaluation. In these cases, fear is more reflexive, driven by deeply ingrained evolutionary mechanisms rather than a conscious assessment of blame or cause. This difference underscores the complexity of fear responses to modern threats, which often require a more nuanced appraisal of both the threat and its broader context.

### 4.2 Role of gender, age, and education in emotional response to ancestral, modern, and pandemic threats

We found that gender, age, and education significantly influenced emotional responses to ancestral, modern, and pandemic threats. Men, older people, and those with biological education reported lower fear and disgust levels toward ancestral and modern fear and disgust elicitors. The role of biological education in moderating emotional responses highlights how specialized knowledge can reduce emotional reactivity by providing a more rational, informed perspective on the risks associated with specific threats (Pinheiro et al., 2016). Women, on the other hand, consistently reported higher levels of fear and disgust across various threat categories, which is consistent with research indicating that they experience stronger fears and anxieties than men (McLean and Anderson, 2009) and suffer more often from phobias like those involving snakes (Polák et al., 2016, 2020).

Our findings also align with research showing that as individuals age, their emotional processing of fear and disgust becomes more nuanced (LoBue et al., 2010) and their disgust sensitivity declines (Fessler and Navarrete, 2005). According to one hypothesis, the cognitive appraisal of disgust changes with age, as individuals accumulate life experience and become desensitized to threats that they have encountered repeatedly (Rozin et al., 1999). This desensitization may reduce the emotional salience of disgust triggers, particularly in older people who may prioritize different types of threats, such as those related to personal safety or health.

Another factor contributing to lower disgust sensitivity with age could be the biological changes that occur in sensory perception, particularly in taste and smell. These are crucial to the disgust response and their decline may impair the ability to detect potentially harmful substances, which could lead to a decrease in the intensity of disgust reactions (Olofsson et al., 2021). In other words, this decline in sensory acuity means that older individuals may no longer receive the same visceral cues that trigger disgust in younger people. However, in our study, participants evaluated written vignettes rather than relying on direct sensory input, which suggests that even cognitive representations of disgust-inducing scenarios are less impactful for older adults. This may reflect age-related changes in how disgust is cognitively processed, moving from a more visceral, immediate reaction in youth to a more abstract, reflective one in older age (Haidt et al., 1994).

Although older respondents generally reported lower levels of fear and disgust across all threat categories, there was one notable exception: pandemic-related fear. In this case, we found a positive correlation between age and fear ratings, with older individuals reporting significantly higher levels of fear compared to younger participants. These findings indicate that older individuals are somewhat failed by the ancient mechanism of the behavioral immune system and disgust as its core component, which is believed to be an evolutionary defense against disease (Oaten et al., 2009). However, as we show here, the human brain might switch to a different emotional response in older age, particularly in the context of pandemic threats. As aging increases vulnerability to illness and mortality, older adults may rely more heavily on fear as a defense mechanism, even though their disgust response wanes. This shift from disgust to fear could reflect an adaptive recalibration in emotional processing, where the recognition of an existential threat (such as illness) becomes more critical than the immediate aversion to potentially contaminating stimuli.

Thus, our results demonstrate that the same threatening stimulus can evoke distinct emotional responses depending on how serious a danger it poses to the individual according to his gender or age category. This finding aligns with the imminent threat theory (Mobbs et al., 2015), which suggests that neural emotional systems are adapted to respond to threats that are most salient and dangerous based on individual characteristics. For example, women’s heightened sensitivity to fear and disgust, especially regarding contamination threats, may have evolved as an adaptive mechanism to protect against potential harm during reproductive years (Kaňková et al., 2023).

### 4.3 Variability of fear, disgust, and anger evaluation explained by individual emotional sensitivity

Multivariate models that accounted for demographic characteristics (gender, age, and education), in addition to the individual’s emotional sensitivity to various threats measured by standard assessments, indicated that while the influence of demographic factors is relatively minor, individual sensitivity plays a crucial role. The Redundancy Analysis (RDA) models revealed significant relationships between the level of evoked fear, disgust, and anger and key psychological factors such as general emotional sensitivity, pandemic-related behaviors, and self-reported fear or disgust of specific stimuli (snakes and toxicity). The first notable result is the overall explanatory power of the RDA models, which accounted for 27.2% of the variance in fear ratings, 22.9% in disgust ratings, and 13.3% in anger ratings. Although the variance explained is moderate, the significance of the findings points to the robustness of these models in capturing meaningful emotional dynamics in response to diverse threat scenarios.

The RDA model for fear ratings demonstrated that pandemic-related behaviors (as assessed by the CSBS) were the most significant predictor suggesting that recent experiences during the COVID-19 pandemic have left a lasting emotional imprint. This supports previous studies indicating that stressful and fear-inducing experiences during pandemics can amplify emotional reactivity to future threats (Pakpour et al., 2021; Şimşir et al., 2022). Fear of specific threats (e.g., snakes, toxicity) was another significant factor influencing fear ratings. This association highlights the role of evolutionary-based fears, where ancestral threats such as snakes continue to evoke strong fear responses (Polák et al., 2016, 2020). However, the inclusion of modern threats, such as toxic substances, shows that emotional reactions are adaptable and can extend to new dangers (Shapouri et al., 2023; Peléšková et al., 2024).

The RDA analyses also suggest that the SNAQ-12 captures both fear and disgust toward vignettes from the ancestral threat category involving snakes. In all the analyses, the snake vignettes are grouped and placed in the multivariate space separately from the rest of the stimuli (see Figure 5) highlighting the specificity of snakes as potent emotional triggers. Interestingly, we observed that this emotional response to snakes is robust and does not vary significantly with the context presented in the vignettes and is unchanged by personal, situational, and environmental factors, despite the relatively low real danger snakes pose today; for instance, no fatal snakebite envenoming has been recorded in the Czech Republic over the past 20 years, while hundreds of individuals die annually in car accidents.

This is in line with previous research that posits that human response to snakes, ancient predators of primates, is deeply rooted in our evolutionary mechanisms for survival and individual experiences or situational factors may have little to no influence (Öhman and Mineka, 2001; Isbell, 2006). The role of both fear and disgust in reactions to snakes further supports the notion that these two emotions are intertwined when facing ancestral threats, with disgust functioning as a contamination-avoidance mechanism and fear triggering immediate defensive behaviors (Curtis et al., 2004; LeDoux, 2012).

While there are well-established instruments for assessing fear elicited by ancestral threats (e.g., people, snakes, heights, enclosed spaces) or infectious diseases (e.g., COVID-19), our study suggests that existing tools may not adequately capture the complexity of emotional responses to modern dangers, such as environmental degradation, radioactivity, or toxic exposure. Given the significant impact of modern threats on public health and psychological well-being, there is an urgent need for the development of new assessment instruments that are specifically tailored to these types of risks. Future scales should be designed to capture the unique characteristics of modern threats - such as their abstract, often delayed nature - and how these characteristics interact with individual sensitivities to generate fear and anxiety.

The RDA results for disgust responses revealed that pathogen disgust (as measured by the TDDS) and disgust toward snakes and toxicity were the most influential variables. This suggests that pathogen-related concerns remain a fundamental driver of disgust, in line with the evolutionary theory of the behavioral immune system (Curtis et al., 2004; Tybur et al., 2016). Interestingly, the second multivariate axis (RDA2) in the disgust model confirmed again a significant negative correlation between age and disgust levels as discussed earlier. This decline in disgust, particularly in response to pathogen threats, raises questions about whether older individuals might be less responsive to cues of contamination or illness, potentially increasing their vulnerability to health risks (Díaz et al., 2020).

One of the most intriguing findings from our analyses is the significant role of pandemic-specific behaviors as measured by the CSBS in predicting fear responses to pandemic-related scenarios. In fact, safety behaviors during the pandemic such as washing hands or checking the internet for information on the virus are a more reliable predictor of emotional responses than the general anxiety level assessed through a methodologically sound measure such as the STAI-X2. While general anxiety is typically viewed as a stable trait that influences emotional sensitivity across different situations (see Dong et al., 2022 for a review), our study suggests that it may not be as predictive in highly specific, high-stress situations like a pandemic.

## 5 Conclusion

An important contribution of this study based on a large data set is its exploration of whether pandemic-related emotional responses more closely resemble ancestral or modern threat reactions. In conclusion, our findings suggest that while pandemic fear is more complex, spanning both ancestral and modern fear categories, pandemic disgust is strongly aligned with ancestral types of disgust-related threats. This supports the hypothesis that the behavioral immune system and survival circuits evolved to respond to infectious diseases are still active in the modern world, particularly in the face of pandemics. In this perspective, the decline in disgust sensitivity as people age raises concerns about older adults’ reduced sensitivity to contamination, potentially increasing their health vulnerability. Further research should explore how these emotional responses evolve over time and in different cultural contexts, particularly in relation to ongoing global threats like pandemics and environmental crises.

Our study highlights that humans employ two key emotions to deal with threats: fear and disgust. They both function effectively but are engaged to varying degrees. Fear is more context-dependent and assesses the immediacy of danger as evidenced by the higher fear ratings associated with modern risks such as car accidents and electricity. Disgust, on the other hand, operates more on an evolutionarily hardwired basis, rooted in ancestral responses, rendering it less effective in addressing contemporary threats such as pollution, toxicity, or radioactivity. When disgust is no longer sufficient as a protective response, fear takes over, especially in older individuals, where disgust sensitivity diminishes with age, reducing its effectiveness in guarding against potential infections.

One of the most interesting findings from our study was that fear of a pandemic is better predicted by an assessment of specific pandemic-related behaviors rather than general anxiety measures. While well-established tools exist for assessing fear related to ancestral and infectious disease threats, our findings suggest these instruments may not fully capture the complexity of emotional reactions to modern dangers, such as environmental degradation or toxic exposure. The results emphasize the need for new assessment tools tailored to these risks, which often have abstract and delayed effects.

## 6 Limitations

One of the key limitations of this study is its cross-sectional design, which may introduce generational effects rather than purely age-related changes. Differences between generations in terms of cultural exposure, environmental conditions, and life experiences are likely to influence emotional responses. Therefore, the observed decrease in disgust and increase in fear or anger among older adults might reflect not only age-related changes in emotional processing but also cohort differences. Longitudinal studies would be valuable to disentangle these effects and provide a clearer understanding of how emotional responses to threats evolve over the lifespan.

## Data Availability

The original contributions presented in the study are included in the article/Supplementary material, further inquiries can be directed to the corresponding author.
