# Supplementary figures and images for: Imprint of ancestral and modern threats in human mind – experience of fear, disgust, and anger
Source: Front Psychol. 2025 Jan 15;15:1520224. doi: 10.3389/fpsyg.2024.1520224 (PMC11774860; doi:10.3389/fpsyg.2024.1520224)

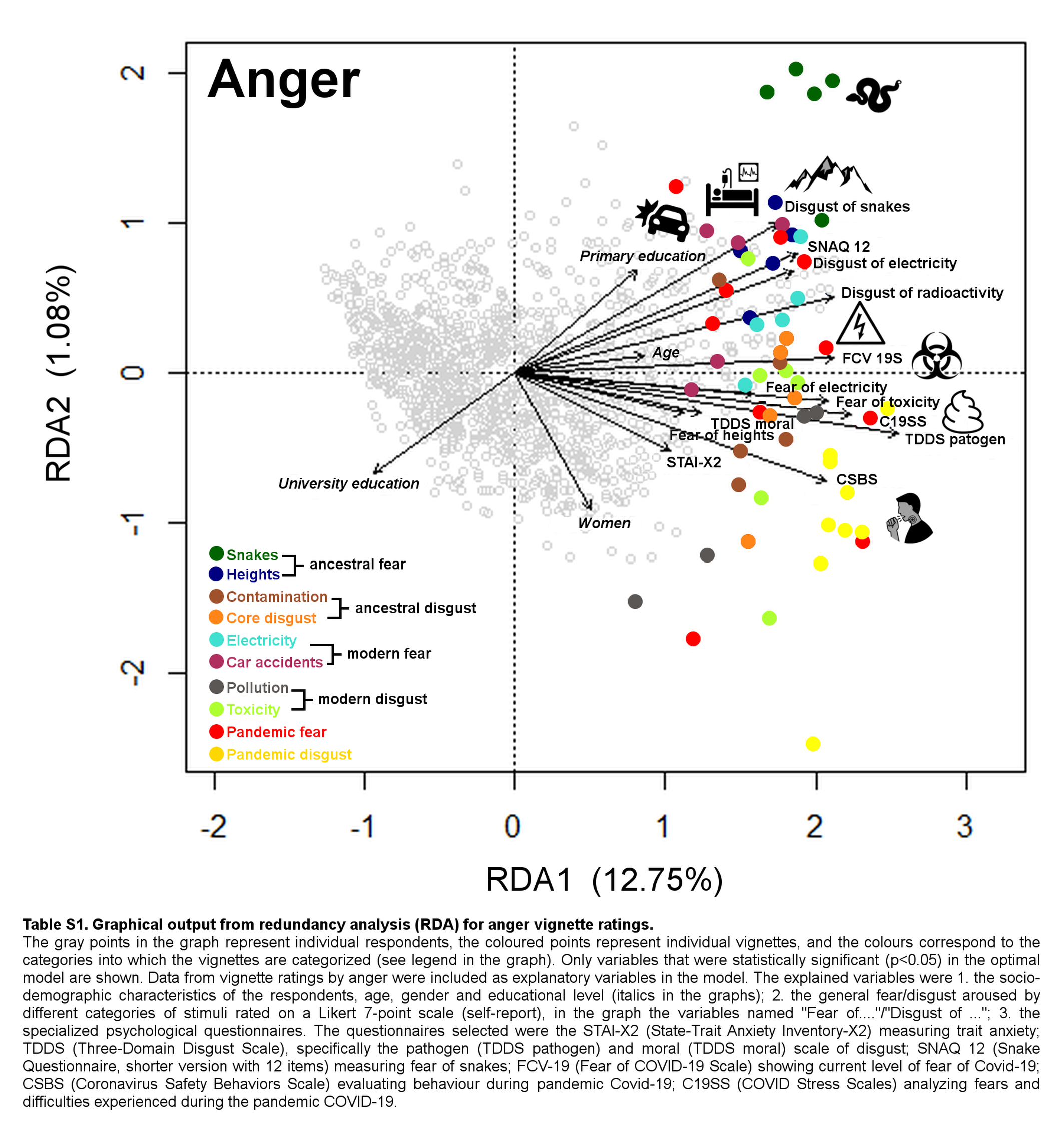

Supplement: Supplementary file 1 [file Image_1.tif]
